# Determination of dialkyl phthalate esters in indoor air of PVC industry: Risk assessment for human health using Monte-Carlo simulations

**DOI:** 10.1016/j.heliyon.2024.e35097

**Published:** 2024-07-25

**Authors:** Shahnaz Sargazi, Ramazan Mirzaei, Mahdi Mohammadi, Mashaallah Rahmani

**Affiliations:** a Health Promotion Research Center, Zahedan University of Medical Sciences, Zahedan, Iran; b Environmental Sciences and Technology Research, Center, Department of Environmental Health Engineering, Shahid Sadoughi University of Medical Sciences, Yazd, Iran; c Social Determinants of Health Research Center, Mashhad University of Medical Sciences, Mashhad, Iran; d Department of Chemistry, Faculty of Sciences, University of Sistan and Baluchestan, Zahedan, 98135-674, Iran

**Keywords:** Dialkyl phthalate esters, Indoor, Environment, Risk assessment

## Abstract

Dialkyl phthalate esters are incorporated to enhance the pliability and prevent brittleness in polyvinyl chloride (PVC) tubing. Exposure to these compounds occurs throughout human lifetimes via ingestion, inhalation, and direct skin contact. A study was conducted to evaluate concentrations of four specific phthalates—dimethyl phthalate (DMP), diethyl phthalate (DEP), di-n-butyl phthalate (DBP), and di(2-ethylhexyl) phthalate (DEHP)—in the indoor air of both industrial and administrative sectors within the PVC manufacturing facilities. Air sampling was conducted in the spring season at two polyethylene factories in Zahedan Industrial Park (Sistan and Baluchestan Province, Iran). The outcomes demonstrated that mean concentrations of these substances in industrial along with administrative departments 485.7 μg/m^3^ and 49.83 μg/m^3^for DMP, 807.38 μg/m^3^ and 30.17 μg/m^3^ for DEP, 849.62 μg/m^3^ and 37.50 μg/m^3^ for DBP along with 1268.08 μg/m^3^ and 45.50 μg/m^3^ for DEHP respectively. The probabilistic lifetime cancer risk (LTCR) of DEHP in the indoor air of Zahedan PVC factories for men and women was determined using the Monte Carlo simulation technique. The computed mean LTCRs of DEHP for men and women in the indoor air of industrial and administrative departments in Zahedan PVC were 1.3 × 10^−3^, 1.2 × 10^−3^and 4.7 × 10^−5^,4.2 × 10^−5^respectively. Data showed that DEHP was a potential risk to human health.

## 1 Introduction

Dialkyl phthalate esters or phthalate acid esters (PAEs) represent a category of organic semi-volatile substances extensively employed as plasticizing agents across a vast array of domestic and industrial applications. Annually, the worldwide production of PAEs exceeds 470 million pounds [1]. Due to their distinctive physicochemical characteristics, PAEs are present in many products, such as floorings made of the PVC, building supplies, personal care items, packaging of food, cleaning agents, and solvents [2,3]. PAEs with low molecular weights, such as dimethyl phthalate (DMP) and diethyl phthalate (DEP), are predominantly utilized in the formulation of cosmetics, personal hygiene products, and surface coatings. PAEs characterized by their high molecular weight, including di(2-ethylhexyl) phthalate (DEHP) along with butyl benzyl phthalate (BBP), are primarily employed in the manufacture of PVC products [1,4]. PAEs can easily be released into the environment, because there is no covalent bond between the phthalates and the plastic they are combined with. So chemical, exposure to PAEs can be created from various sources, such as water, air, dust, soil, packaged foods and, the use of personal and consumer care products [[5], [6], [7], [8]]. As early as the 1960s, PAEs toxicity started to garner public notice. The United States Environmental Protection Agency (EPA) and a few additional countries have identified the most frequent PAEs as priority pollutants [5]. PAEs can produce dermatological problems and affect several aspects of human health, especially the reproductive, respiratory, and endocrine systems [9]. Therefore, a report on PAEs in urban atmosphere has been of great importance for addressing environmental and human health issues [10]. For instance, research indicated that the aggregate levels of PAEs detected in office and residential structures across China are estimated at roughly 3800 (ng/m^3^) [11]. In comparison, markedly lower concentrations of PAEs have been observed in domestic environments in France, at approximately 320 (ng/m^3^), and in North America, where the levels were around 540 (ng/m^3^) [12,13]. Statistical analysis was indicated that DEHP the predominant phthalate ester present in indoor air, accounting for 40.6 % of the overall phthalate esters detected. This is followed by DMP, DEP, DBP, butyl benzyl phthalate (BBP), and di-n-octyl phthalate (DnOP), with each constituting between 9 % and 15 % of the total phthalate esters recorded [14]. Alternatively, indoor sources of PAEs consist of construction materials and interior furnishings, along with consumer products, constituting a significant proportion of the phthalates to which individuals are exposed [15].

Studies on occupational PAEs exposure are relatively limited. Among commercial PAEs, DEHP is the primary phthalate plasticizer for the PVC [[16], [17], [18]]. More than two million tons of DEHP are applied worldwide each year. Its potential health and environmental risks cannot be ignored due to its low degradability [19]. This chemical is endocrine-disrupting, and carcinogenic [16,20,21] and EPA has been added DEHP to the list of "Chemicals of Concern" [22].

The existence of various industries in Iran and the need for the PVC products in Iran and the world in such a way that the annual demand for the PVC around the world is stated to be approximately 35 million tons [23] justifies the need for such research. Furthermore, in recent years, most occupational research has been focused on DEHP compound [16,24,25], and lighter compounds such as DMP, DEP, and DBP have been neglected. Nevertheless, this study has investigated the occurrence of four phthalates (DMP, DEP, DBP, and DEHP) in indoor air of administrative and industrial departments in Zahedan PVC industries (Sistan and Baluchestan Province, Iran). LTCR calculated the probabilistic of DEHP in two environments using the Monte Carlo simulation technique and Hazard Quotients (HQ) were calculated the DMP, DEP, DBP, and DEHP compounds.

## 2 Materials and methods

### 2.1 Air sampling

Four phthalate esters that contaminated indoor air in Zahedan city in the SouthEast of the Islamic Republic of Iran PVC factories were studied. Two PVC factories were selected in the Industrial Park. The ventilation system was installed at the beginning and end of the production hall.

Air samples were collected following the methodology outlined in the OSHA Manual of Analytical Methods, procedure number 104 [20]. To capture air samples, personal sampling pumps from SKC, fitted with a variable low-flow holder, were deployed. Calibration of flow rates was meticulously achieved through the utilization of a soap bubble flow meter. The air sampling process was executed at a consistent flow rate of 1 L per minute, extending for 8 h, ultimately amounting to a cumulative air volume of 480 L sampled. To collect of indoor air PAEs, Tenax OVS sorbent tubes provided by SKC were employed as the primary sampling medium. Sampling was carried out in areas where workers were most commuting and monitored for PAEs compounds in the spring season (n = 16). Air sampling was conducted by collecting samples from the typical inhalation area of personnel, approximately 150 cm above the floor. These samples were meticulously preserved at a temperature of −20 °C before undergoing analysis evaluation.

### 2.2 Preparation and analysis of air samples

For sample preparation and analysis first, the plastic inlet and outlet were separated from the Tenax and the front, and back sections of the Tenax sorbent tubes were spilled into two separate vials. Then 4 ml of the toluene solvent into any Tenax absorbent glass container was poured and shaken for 30 min until the extraction was carried out into the solvent [20].

**Fig. 1:**
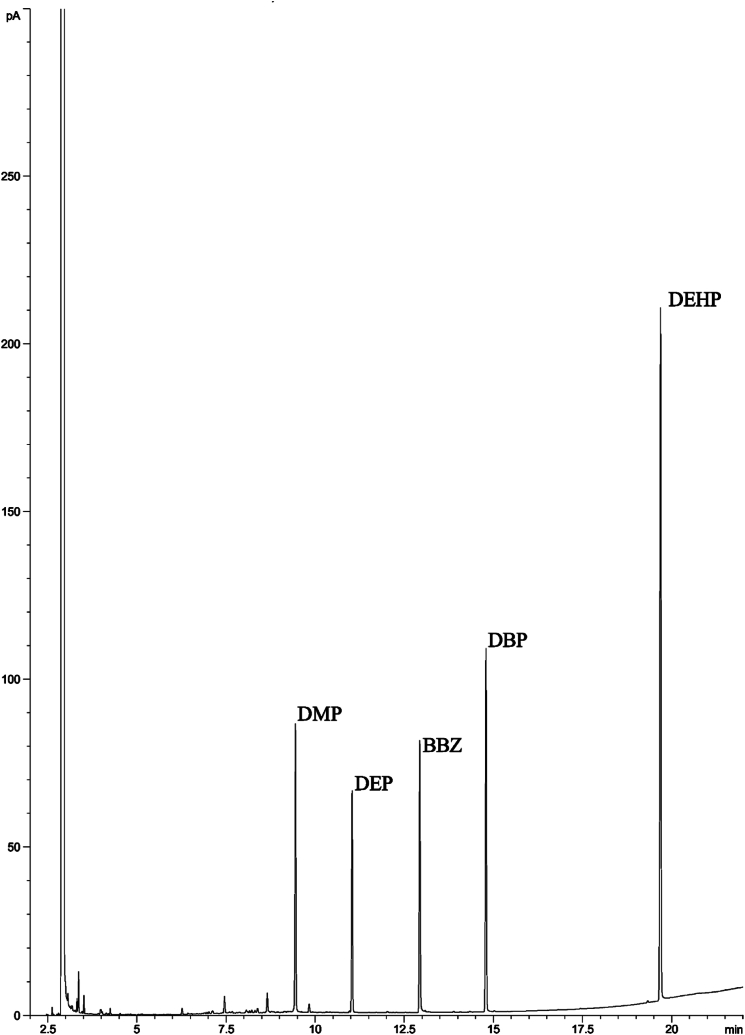
Typical chromatograms of an air sample from a breathing zone in a PVC factory. DMP, DEP, BBZ, DBP, and DEHP concentration: 28, 99, 34, and 144 μg/L, respectively. BBZ (internal standard).

To prepare the standard curve, the standard solutions were injected into the gas chromatograph and the calibration curve was plotted according to the sample concentration, and calculated peak area. The solvent underwent a transfer into vials suitable for gas chromatography (GC) analysis, and the quantities of PAEs compounds were determined using an Agilent 7890A GC system. This system was fitted with a Flame Ionization Detector (FID) and an HP-5 Agilent fused-silica capillary column, measuring 30 mm long, with an internal diameter of 0.32 mm and a 0.25 μm film thickness. The temperatures for both the injector and the detector were precisely set to 270 °C and 275 °C, respectively. The oven temperature was programmed at 90 °C for 0.5 min and then 10 °C/min to 290 °C. Under these conditions, for a run time of 22.5 min, retention times were 9.4, 11.1, 12.9, 14.8, and 19.7, for DMP, DEP, BBZ, DBP, and DEHP, respectively (Fig. 1).

The gas chromatography detector underwent a rigorous calibration process involving five standard solutions, each containing varying concentrations of PAEs from 10 to 500 μg per liter. The calibration curves for DMP, DEP, DBP, and DEHP exhibited strong linear relationships with R-squared values of 0.992, 0.988, 0.992, and 0.996, respectively. To ensure precision and accuracy throughout the analytical series, one of these calibration standards was included in each batch of samples. Moreover, the quantification of PAEs was carried out using an internal standard method to bolster the reliability of the measurement outcomes.

### 2.4 Health risk assessment

The assessment of both non-carcinogenic and carcinogenic risks posed by PAEs was carried out under strategies endorsed by the US EPA. Within the spectrum of PAEs compounds, DMP, DEP, and DBP have been classified as non-carcinogenic concerning human health. However, DEHP has been identified as a potential carcinogen. Given that human exposure to PAEs in the environment predominantly occurs through inhalation, this study is focused solely on evaluating the inhalation exposure routes. The average daily dose (ADD) for non-carcinogenic exposure and the lifetime average daily dose (LADD) for carcinogenic exposure were quantified using Equation (1), [26].

(1) $$ADD\;orLADD=\frac{c\times IR\times EF\times ED\times CF}{BW\times AT}$$

The metrics employed for assessing the Acceptable Daily Dose (ADD) and the Lifetime Average Daily Dose (LADD) are denoted in mg/kg-day. The variable 'c' represents the total concentration of phthalates in the atmosphere, expressed in μg/m^3^. The term 'IR' illustrates the inhalation rate, quantified in m^3^/d. The exposure frequency is indicated by 'EF' and is measured in days/year, while 'ED' signifies the exposure duration, denoted in a year. 'BW' stands for body weight and is calculated in kg. The 'AT' parameter indicates the average lifetime, which is measured in days, and 'CF' refers to the conversion factor required to convert from mg/μg.

For non-carcinogenic compounds, the Hazard Quotient (HQ) is determined by utilizing Equation (2) as follows.

(2) $$\text{HQ}=\frac{\text{ADD}}{\text{RfC}}$$

The HQ is a numerical value used to evaluate the potential risk of exposure to hazardous substances. It is calculated by comparing the expected level of exposure with a safe reference point known as the Reference Concentration (RfC). The RfC represents an estimated threshold for chronic inhalation exposure that the general population, including vulnerable subgroups, can sustain for a lifetime without experiencing harmful effects. The RfC values for DMP, DEP, DBP, and DEHP are depicted in Table 1 [24,27]. For cancer compounds, LTCR is calculated with the following Eq. (3).

(3) LTCR=LADD×SF

where LTCR is a parameter that quantifies the risk of developing cancer throughout an individual's lifetime. It is associated with exposure to carcinogenic substances. The Carcinogenic Slope Factor (SF) is a critical component in calculating LTCR and represents the increased cancer risk per unit of exposure to a carcinogen. For DEHP, the SF has been determined to be 0.014 kg-day/mg, which denotes the estimated risk associated with daily exposure to DEHP over a lifetime [24]. The values of the parameters related to the calculation of LTCR and HQ are given in Table 1. Individuals are regarded as being at risk for non-cancer health effects if the Hazard Quotient (HQ) exceeds 1. Conversely, the likelihood of cancer is deemed to be very low or insignificant when the LTCR is at or below the threshold of 10^−4^ [27].

**Table 1:** Human exposure factors through the respiratory system.

parameters	Adult males	Adult females	Reference
Inhalation rate (m^3^/day)	19.02	14.7	[23]
Body weight (kg)	62.7	54.4	[23]
Exposure frequency (day/year)	261	261	[23]
Exposure duration (year)	24	24	[23]
aAveraging time (non-cancer)/day	8760	8760	[23]
bAveraging time (cancer)/day	25550	25550	[23]
bInhalation reference concentration of DMP, DEP, DBP and DEHP (bRfC)(mg/kg-day)	0.015 for DMP	0.013 for DMP	[19,24]
	0.85 for DEP	0.76 for DEP	[19,24]
	0.0061 for DBP	0.0054 for DBP	[19,24]
	0.021 for DEHP	0.019for DEHP	[19,24]
Cancer slope factor(mg/kg day)-1 for DEHP	0.014 kg.day/mg	0.014 kg.day/mg	[19]

^c^RfC with the unit mg/kg-day)= (inhalation reference concentration (0.05, 2.8, 0.02, 0.07 mg/m^3^ for DMP, DEP, DBP and DEHP) × Assumed inhalation rate (m^3^/day) × 1/BW (kg.

a Averaging exposure time (days) for Noncarcinogens = (24 years) × 365 days per year.

b Averaging exposure time (days) for carcinogens = (70 years) × 365 days per year.

### 2.5 Statistical analysis

The data were entered into SPSS version 25 (SPSS Inc, Chicago, USA) and then analyzed using descriptive statistics (setting the table, determining statistical indicators) and inferential statistics (correlation coefficient test).

## 3 Results and discussion

### 2.3 Quality control and quality assurance

The analytical method's recovery efficiency was evaluated by adding 100 μg of the specified analytes into clean Tenax tubes. These tubes underwent extraction and analysis processes identical to those applied to field samples. The overall recovery of phthalate esters was found to be exceptionally high, with an average of 99.3 % (ranging from 98.4 to 99.8 %). Additionally, control samples, including eight field blanks and five method blanks, were prepared and analyzed following the established procedures to ensure the method's reliability and consistency.

### 3.1 Evaluation of dialkyl phthalate esters levels in the indoor air of different sections of PVC industries

The levels of PAEs in the indoor air across various sectors of Zahedan's PVC manufacturing and administrative facilities exhibited significant variation. Measurements showed that the aggregate concentrations of PAEs (ΣPAEs) ranged from 46.00 μg/m³ to 8370 μg/m³, with a median value of 3250 μg/m³ and average concentrations of 3410 μg/m³ in industrial areas and 164 μg/m³ in administrative spaces. (Table 2). Based on mean values, DMP, DEP, DBP, and DEHP were equal to 485.79 μg/m^3^,807.38 μg/m^3^, 849.62 μg/m^3^and 1268.08 μg/m^3^ in the indoor air of the industrial section, respectively. The highest amount of pollutants in the workplace was related to DEHP > DBP > DEP > DMP, respectively. In this study, the highest concentration was observed for DEHP (3544 μg/m^3^) and a mean of 1268 μg/m^3^. DEHP values in the industrial environment were almost a little more than half a quarter of the standard value set in the occupational exposure limit (OEL) values (OSHA PEL-TWA: 5 mg/m^3^) [16] (Table 2).

**Table 2:** Statistical summary of PAEs concentrations (μg/m^3^) in the industrial and administrative sections.

Site		DMP	DEP	DBP	DEHP	∑PAEs
Industrial section (n = 16)	Mean	485.79	807.38	849.62	1268.08	3.41 × 10^3^
	Median	278.05	627.65	476.40	708.25	3.25 × 10^3^
	Std. Deviation	496.07	738.43	936.76	1343.53	2.65 × 10^3^
	Range	1555.00	2128.00	2496.00	3507.00	8.37 × 10^3^
	Minimum	28.00	25.00	18.00	37.00	156.00
	Maximum	1583.00	2153.00	2514.00	3544.00	8528.80
Administrative section (n = 6)	Mean	49.83	30.17	37.50	45.50	163.00
	Median	51.50	28.00	36.50	47.50	164.00
	Std. Deviation	17.85	9.85	9.65	6.98	17.79
	Range	45.00	27.00	27.00	16.00	46.00
	Minimum	28.00	18.00	25.00	37.00	139.00
	Maximum	73.00	45.00	52.00	53.00	185.00

Also, in the administrative department, average values of 49.83 μg/m^3^, 30.17 μg/m^3^, 37.50 μg/m^3^ and 45.50 μg/m^3^ were reported for DMP, DEP, DBP, and DEHP compounds (Table 2). Accordingly, in the office environment, the highest values belonged to DMP > DEHP > DBP > DEP, respectively. The values of DMP, and DEHP were reported to be almost a hundred times lower than the standard limit [28].

The levels of PAEs identified in the present study significantly exceed those recorded in the indoor air of similar microenvironments in past research. For instance, the concentrations of PAEs exhibited significant variability, ranging from 223 to 6176 ng/m^3^ in homes [12,13,16,[29], [30], [31]], 3590–4748 ng/m^3^ in offices along with apartments [11,17,32], to 0.80 × 10^3^ to 6.4 × 10^3^ ng/m^3^ [26]in laboratories. Notably, in some laboratories is identified PAEs levels of up to 6.39 × 10^4^ ng/m^3^. The dominant PAEs, including Diisobutyl phthalate (DiBP), di(methoxyethyl) phthalate (DMEP), and DBP, demonstrated median levels of 0.48 × 10^3^, 0.44 × 10^3^, and 0.39 × 10^3^ ng/m^3^, respectively, followed by di-(2-propylheptyl) phthalate (DPHP) along with DEHP with median levels of 0.16 × 10^3^ and 0.13 × 10^3^ ng/m^3^, respectively. In Toronto, Canada, a comprehensive study was conducted to assess the nail salon employees' exposure to ten phthalates in 18 nail salons. During an approximate 8-h working period, 45 nail salon workers wore silicone passive samplers, such as wristbands (n = 60) and brooches (n = 58), as well as active air samplers (n = 60). The study revealed that DEP and diisobutyl phthalate with a median 471 ng/m^3^ and 337 ng/m^3^ exhibited the highest levels in the active air samples [33].

The report's methodology involved the utilization of the Spearman correlation matrix to identify strong correlations between pairs of PAEs and to evaluate the origin of each pair of individual PAEs. (Table 3). In industrial buildings some pairs of PAEs had strong correlations, such as DMP versus DBP (r = 0.762) and DMP versus DEHP (r = 0.606). DBP and DEHP were two of the most used PAEs in commercial products and were listed as hazardous pollutants by the US EPA. The compelling evidence of strong correlations between high-molecular-weight PAEs, particularly DBP and DEHP, strongly suggests a shared origin in air samples collected from the indoor air of industrial building areas [34].

**Table 3:** Spearman correlation matrix for PAEs.


			DMP	DBP	DEHP
Spearman's rho	DMP	Correlation Coefficient	1.000	0.762**	0.606*
		Sig. (2-tailed)	.	0.001	0.013
		N	16	16	16
	DBP	Correlation Coefficient	0.762**	1.000	0.471
		Sig. (2-tailed)	0.001	.	0.066
		N	16	16	16
	DEHP	Correlation Coefficient	0.606*	0.471	1.000
		Sig. (2-tailed)	0.013	0.066	.
		N	16	16	16
**. Correlation is significant at the 0.01 level (2-tailed).					
*. Correlation is significant at the 0.05 level (2-tailed).					

### 3.2 Sources of indoor dialkyl phthalate esters

The sources of indoor PAEs are associated with decoration characteristics, lifestyles, and working conditions [35,36]. Variations in the types and concentration levels of PAEs in different compartments are attributed to differences in pollution sources and other environmental factors in various microenvironments [16]. The discrepancy in PAEs concentrations between industrial buildings and offices may be due to the proximity of sampling locations to hot spots where PVC pipes are manufactured. Due to their high temperature, these hot spots cause easy release of DEHP compound in the factory air. Additionally, differences in ventilation may be a factor. Incomplete mechanical ventilation in the industrial environment causes the accumulation of air in the environment and increases the concentration of pollutants such as DEHP and DBP.

The primary sources of PAEs in office buildings are the flooring and the percentage of plastic materials in furniture, decoration, and electronic devices [16]. Moreover, the mechanical ventilation of office environments through air ducts may facilitate the dilution and exhaust of indoor phthalate esters [37,38]. The presence and fate of PAEs in indoor environments are attributed to indoor air volume, leachability, humidity, rate of air exchange between the indoor and outdoor environments, features of the building, and indoor temperature [39,40]. With outdoor air, wind direction was also found to impact the air quality inside the building significantly [34].

### 3.3 Health risk assessment

**Fig. 2:**
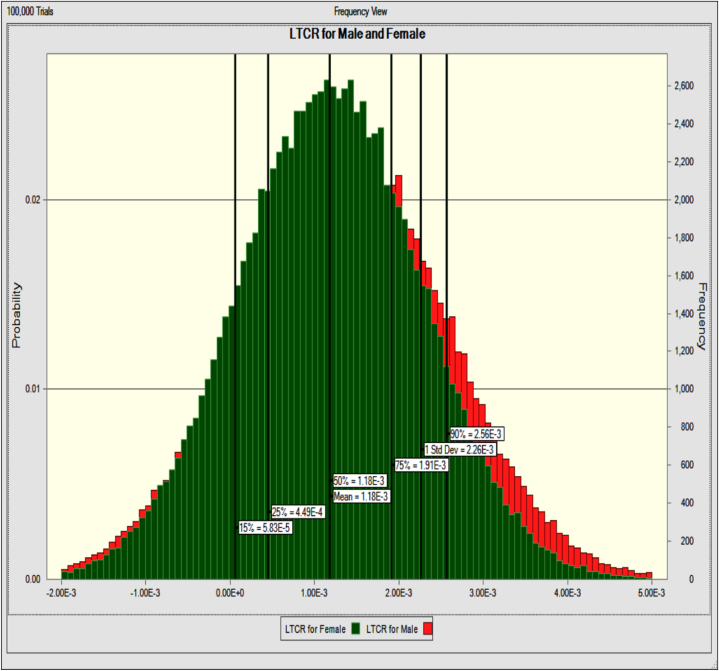
The Monte Carlo simulation method for the probabilistic LTCR of DEHP in the indoor air of Zahedan PVC plants for both men and women.

The probabilistic LTCR of DEHP in the indoor air of Zahedan PVC factories for men and women was determined using the Monte Carlo simulation technique, as shown in Fig. 2. A total of 100,000 trials were performed to investigate the risk, and the total DEHP was utilized to calculate the LTCR. The computed mean LTCRs for men and women in the indoor air of industrial and administrative departments in Zahedan PVC were 1.3 × 10^−3^, 1.2 × 10^−3^and 4.7 × 10^−5^,4.2 × 10^−5^respectively. This represents an elevated risk compared to a desirable baseline level (1E- 06). According to international supervisory organizations, LTCR values below 10^−6^ suggest allowed safety, between 10^−6^, and 10^−4^ indicate a potential danger, and beyond 10^−4^ indicate a severe potential hazard. Nevertheless, in the industrial sector, the potential risk is severe and the administrative sector shows a potential risk [34].

The probability density curves for the LTCR combined amounts of DEHP in men and women are shown in Fig. 2. The Monte Carlo simulation's predefined percentiles (15th, 25th, 50th, 75th and 90th) are used to represent the risk distribution for both men and women. Probability distributions for both genders have shown that observations are biased toward high-risk values. The USEPA's recommended acceptable exposure threshold is 10^−6^ [22], while the 15th percentile for men and women showed an exposure of roughly 58 times higher. Therefore, for both groups, the likelihood of cancer after inhaling DEHP was >10^−6^ in 90 % of instances. Since the result exceeded the baseline value, the 15th percentile value for both men and women indicates the risk of cancer (10^−6^). In the PVC industries department of Zahedan, men and women are likely to get cancer because the 25th and values at the 50th percentile of the total risk of cancer are, respectively, about 449 and 1180 times greater than the allowable value (1E-06). The 75th along with 90th percentile values indicate that the risk for both men and women would be increased by 1910 and 2560 times, respectively. Fig. 2 showed that the indoor air of Zahedan PVC industries was carcinogenic to men and women.

Hazard Quotient values, including DMP, DEP, DBP, and DEHP for men and women in this data set, ranged from 0.4 to 22.6, from 0.0046 to 0.55, from 0.64 to 89.9 and from 0.37 to 12.9 in PVC industries department and administrative department, respectively. The results of the study are summarized in Table 4. This data was reported as different in level compared to some previous studies [7,8,21]. HQ values higher than one indicate severe exposure circumstances linked to an elevated risk of chronic non-cancerous illness in the targeted human organs. To lower PAEs concentrations to a safe and healthy level, the interior air exchange rate must be significantly increased [41]. Due to the considerable non-carcinogenic dangers connected to PAEs, it is necessary to establish proper health warning labels, regulations addressing indoor to reduce the risks to people's health in these locations and, using adequate and appropriate safety equipment to prevent excessive exposure in industrial environments. Overall, the research has provided basic scientific data that may help environmental management agencies issue regulations to control these pollutants in the future [34]. It should also be noted that this study has not considered other potentially significant intake routes, such as meal ingestion, settling dust ingestion, occupational exposure, and so forth [16].

**Table 4:** The PAEs' non-cancer risk factors HQ.

PAEs compounds	Persons	Industrial section	Administrative section
DMP	Adult males	6.95	0.71
	Adult females	2.39	0.71
DEP	Adult males	0.21	0.008
	Adult females	0.21	0.008
DBP	Adult males	30.38	1.34
	Adult females	2.21	1.34
DEHP	Adult males	12.95	0.46
	Adult females	12.95	0.46

Eventually, more concrete recommendations were presented on potential intervention strategies to mitigate the identified risks of phthalate exposure in the PVC manufacturing facilities:

Engineering controls (substitution of materials, enclosure and ventilation, closed systems), personal protective equipment (respiratory protection, skin protection, eye protection), work practices and procedures (safe handling protocols, training and education, regular maintenance), monitoring and exposure assessment (air monitoring, biological monitoring), hazard communication (labeling and signage, material safety data sheets), waste management (segregation and disposal, recycling alternatives), continuous improvement (risk assessment, feedback mechanisms). By implementing these intervention strategies, the PVC manufacturing facilities can effectively mitigate the risks associated with PAEs exposure, safeguarding the health and well-being of workers and ensuring compliance with regulatory requirements.

## 4 Limitations

One of the study's shortcomings was the absence of availability to a more significant number of PAEs group analytes and monitoring of environmental parameters, which affected the research results and was not presented as intended. For a review of these items, financial, as well as timing constraints, prevented it from happening.

## 5 Conclusions

An overview of the levels of PAEs in indoor air of the industries department, and administrative department at the PVC factories is given by this study. It also examined the environmental health risks that these substances may pose. All four investigated chemicals were found in the 163–3.41 × 10^3^ μg/m^3^ range of the air samples. The highest amount of pollutants in the workplace was related to DEHP > DBP > DEP > DMP, respectively. But in the office environment, the highest values belonged to DMP > DEHP > DBP > DEP, respectively. The assessment of the health risk of PAEs in the indoor air of the industrial sector demonstrated that the potential risk is severe, and the administrative sector indicates a potential risk. As a result, this is a significant problem that needs to be handled with wise management choices.

## Data availability statement

All data generated or analyzed during this study are included in this manuscript and its supplementary information files. The data that support the findings of this study are available on request from the corresponding author. The data are not publicly available due to privacy.

## CRediT authorship contribution statement

**Shahnaz Sargazi:** Writing – original draft, Software, Methodology, Formal analysis, Conceptualization. **Ramazan Mirzaei:** Writing – review & editing, Methodology, Formal analysis, Conceptualization. **Mahdi Mohammadi:** Writing – review & editing, Data curation, Conceptualization. **Mashaallah Rahmani:** Writing – review & editing, Software, Methodology, Data curation, Conceptualization.

## Declaration of competing interest

The authors declare that they have no known competing financial interests or personal relationships that could have appeared to influence the work reported in this paper.
